# A Rare Case of Nasal Rhinosporidiosis in the Santhal Pargana Region of Jharkhand: Clinical Presentation and Management

**DOI:** 10.7759/cureus.72674

**Published:** 2024-10-30

**Authors:** Gulistan Bano, Sumeet Angral, KSBS Krishna Sasanka, Saurabh Varshney, Pradosh Kumar Sarangi

**Affiliations:** 1 Otorhinolaryngology-Head and Neck Surgery, All India Institute of Medical Sciences, Deoghar, Deoghar, IND; 2 Otorhinolaryngology-Head and Neck Surgery, All India Institute of Medical Sciences, Bilaspur, Bilaspur, IND; 3 Radiodiagnosis, All India Institute of Medical Sciences, Deoghar, Deoghar, IND

**Keywords:** jharkhand, nasal mass, rhinosporidiosis, rhinosporidium seeberi, santhal pargana, surgical excision

## Abstract

Rhinosporidiosis is a chronic granulomatous disease affecting mucosal membranes, particularly in tropical and subtropical regions. This case report presents a young male from Santhal Pargana, Jharkhand, who presented with nasal obstruction, epistaxis, and a mass in the right nasal cavity. Surgical excision and histopathological analysis confirmed rhinosporidiosis. Although no definitive medical treatment exists, surgical excision with cauterization remains the most effective method to prevent recurrence. This report marks the first documentation of nasal rhinosporidiosis in Santhal Pargana, Jharkhand.

## Introduction

Rhinosporidiosis, caused by *Rhinosporidium seeberi*, is a chronic granulomatous disease primarily affecting the nasal mucosa and nasopharynx [1]. It is prevalent in tropical and subtropical regions, especially in South India and Sri Lanka, although cases have also been reported in the Americas, Europe, and Africa. The disease is endemic in certain states of India, such as Chhattisgarh, Tamil Nadu, Kerala, Orissa, and eastern Madhya Pradesh [2]. The infection is often acquired from contaminated water [3]. It typically presents with nasal obstruction, epistaxis, and rhinorrhea and features friable, vascular masses in the nasal cavity [4]. A definitive diagnosis requires histopathological examination, which reveals large sporangia filled with endospores. Surgical excision with cauterization is the standard treatment, as medical therapy has proven ineffective [5]. This report documents the first case of nasal rhinosporidiosis in the Santhal Pargana region of Jharkhand, highlighting the clinical presentation, diagnostic challenges, and successful surgical management in the non-endemic area. It underscores the importance of recognizing this condition, even in rarely reported regions.

## Case presentation

A 33-year-old man from the Santhal Pargana region of eastern India presented with an eight-month history of right-sided nasal obstruction, exacerbated by cold episodes and unresponsive to decongestants. He reported three to four episodes of mild to moderate nasal bleeding associated with cold exposure, which were managed with nasal pressure and antifibrinolytics. There was no history of swimming, exposure to stagnant water, nasal trauma, or significant systemic illness.

**Figure 1:**
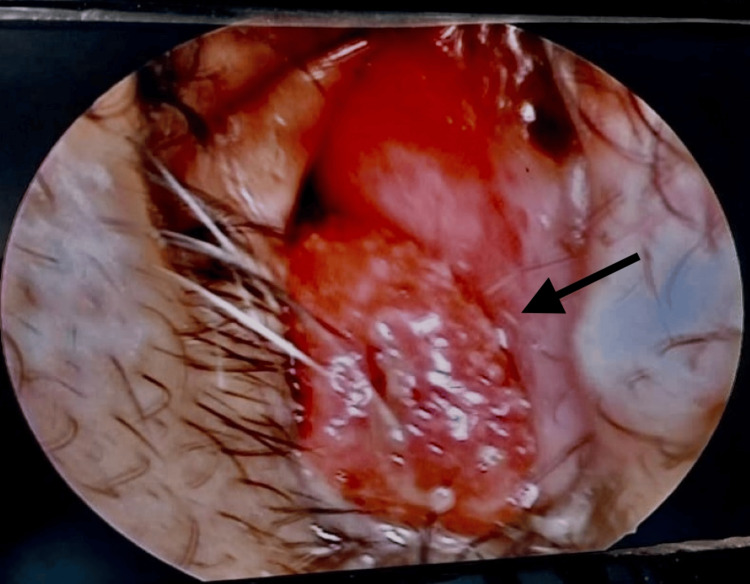
Endoscopic view of the right anterior nasal space showing a reddish, strawberry-like mass mass in the right nasal cavity.

The clinical examination revealed a reddish, strawberry-like mass, approximately 2 x 2 cm in size, located in the inferior meatus of the right nasal cavity, which was better visualized on nasal endoscopy (Figure 1). The mass was friable, non-tender, studded with white dots, mobile with a sessile attachment to the inferior turbinate, and free from the nasal septum, floor, and middle turbinate. No bleeding occurred upon gentle probing. Examination of the left nasal cavity, nasopharynx, oropharynx, ears, and eyes, as well as a systemic head and neck examination, showed no abnormalities.

**Figure 2:**
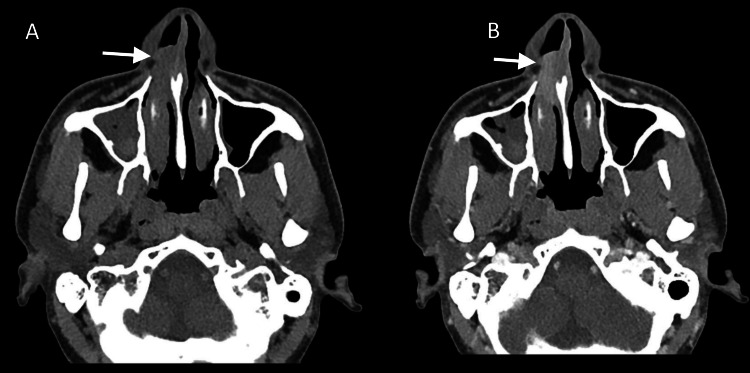
Axial views of non-contrast (A) and contrast-enhanced (B) CT PNS showing an enhancing soft tissue mass lesion in the right inferior nasal cavity (arrow) extending anteriorly into the vestibule. CT: computed tomography; PNS: paranasal sinus.

Initial blood investigations, including routine hematology and biochemistry, were normal. A contrast-enhanced CT (CECT) scan of the nose and paranasal sinuses revealed an enhancing soft tissue mass in the right inferior nasal cavity extending anteriorly into the vestibule, with mucosal thickening in bilateral maxillary sinuses (Figure 2A, B).

**Figure 3:**
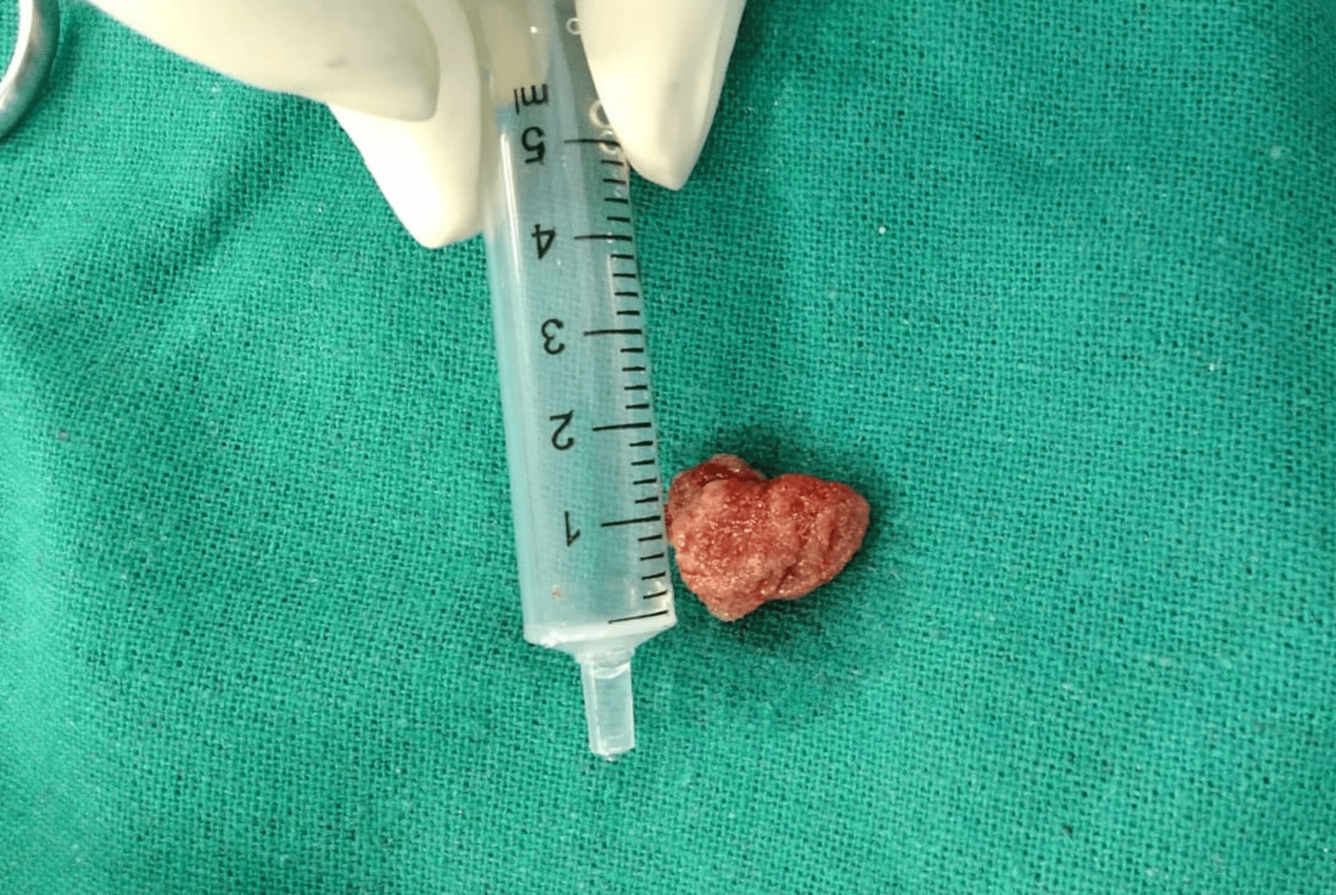
Photograph showing the resected mass (1.5x1.8 cm).

After obtaining informed consent, the patient underwent endoscopic excision of the mass under local anesthesia. The mass was excised en bloc, and the base was cauterized to prevent recurrence. Minimal intraoperative bleeding occurred, eliminating the need for nasal packing. The excised pink, pedunculated mass was sent for histopathological examination (Figure 3).

**Figure 4:**
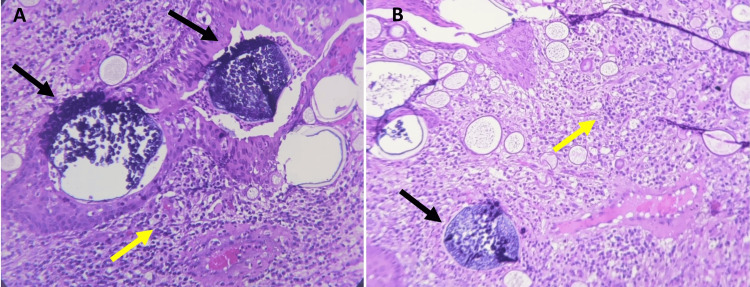
(A,B) Histopathological examination of the right nasal mass showing mature sporangia (black arrows) containing endospores, with sporangia surrounded by mixed chronic inflammatory cells (yellow arrows) (H&E, 100x). H&E: hematoxylin and eosin.

Histopathological analysis confirmed the diagnosis of rhinosporidiosis, revealing numerous large, thick-walled sporangia containing endospores, along with a dense inflammatory infiltrate composed of lymphocytes, plasma cells, neutrophils, and eosinophils (Figure 4A, B). No evidence of malignancy was found in the examined tissue.

**Figure 5:**
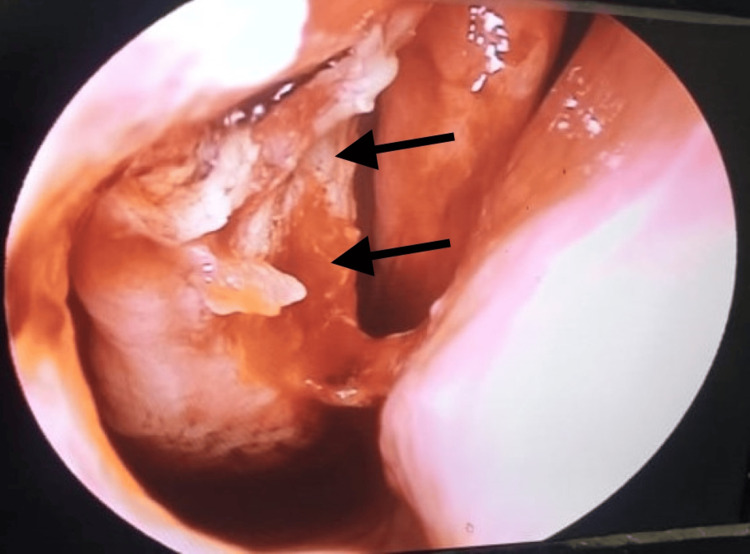
Endoscopic image showing crust formation at the cauterized base of the right inferior turbinate on postoperative day 10.

The patient was discharged on the third postoperative day in stable condition and prescribed oral antibiotics, nasal decongestants, and saline drops. Follow-ups were scheduled weekly for the first month, biweekly for the second, and monthly thereafter. Mild crusting at the cauterized base on day 10 resolved with saline drops (Figure 5). After three months, there were no recurrences or complications, and the patient was advised to avoid stagnant water to prevent reinfection.

## Discussion

Rhinosporidiosis is a rare, chronic granulomatous infection caused by *Rhinosporidium seeberi*, an aquatic protistan parasite with a debated classification. Once thought to be a fungus due to its morphology and positive staining, molecular studies now place it in the class Mesomycetozoea. The disease is endemic in tropical and subtropical regions, especially in South India and Sri Lanka, where infections are linked to exposure to stagnant water. However, cases have also been reported outside endemic areas, often in individuals with a history of travel to those regions. The nose is the primary site of infection, followed by ocular lesions, but it can also affect the nasopharynx, larynx, oropharynx, and genital mucosa [6].

The pathogenesis of rhinosporidiosis involves local replication of *Rhinosporidium seeberi* in mucosal tissues, resulting in hyperplastic growth and friable, polypoid masses with a "strawberry-like" appearance. Visible white dots indicate mature sporangia that release endospores upon rupture, possibly leading to autoinoculation or further spread. Factors such as nonspecific immune reactivity, blood type, human leukocyte antigen (HLA) types, and a shift from Th-1 to Th-2 immunity may influence infection [6,7].

In this case, the patient had no history of pond bathing, swimming, or travel to endemic areas, suggesting possible non-water-related transmission through contaminated soil or dust. The infection appeared localized to the nasal cavity, with no systemic symptoms or involvement of other mucosal sites.

Diagnosis is primarily clinical, supported by histopathology, with the characteristic appearance of sporangia being pathognomonic. Common stains like hematoxylin and eosin (H&E), Gomori methenamine silver (GMS) stain, mucicarmine, and periodic acid-Schiff (PAS), along with advanced methods like PCR or 16S RNA analysis, can aid diagnosis [3,8]. In this case, histopathological examination revealed large sporangia containing endospores, surrounded by chronic inflammatory cells, confirming the diagnosis.

Managing rhinosporidiosis is challenging due to the high risk of recurrence. Medical therapy, including antifungal agents like dapsone, has limited effectiveness; dapsone may inhibit sporangial maturation and promote fibrosis but is not curative. Surgical excision with wide margins and cauterization remains the gold standard [9]. In this case, endoscopic excision achieved complete mass removal, with no recurrence over a one-year follow-up.

The prognosis is generally good after complete surgical excision, but complications such as dissemination to other mucosal sites, local infections, and recurrence can occur. The risk of recurrence is especially high with incomplete excision or trauma to the lesion, which can release endospores into surrounding tissues [10,11].

This case represents the first documented instance of nasal rhinosporidiosis in the Santhal Pargana region of Jharkhand. The rarity of this condition in non-endemic areas emphasizes the need for awareness among clinicians, particularly when evaluating patients with nasal masses. Proper public health education, especially regarding the risks of exposure to contaminated water and soil, may help reduce the incidence of this disease in both endemic and non-endemic regions.

## Conclusions

Rhinosporidiosis should be considered a differential diagnosis in patients presenting with nasal masses, especially in endemic regions. Surgical excision with cauterization remains the most effective treatment. This report represents the first documented case of nasal rhinosporidiosis in the Santhal Pargana region of Jharkhand, highlighting the potential for disease occurrence in previously unreported areas. Awareness of the disease and education about minimizing exposure to potentially contaminated water and soil are critical in reducing the incidence of this infection. Continued research and case reporting are essential to enhance understanding of rhinosporidiosis, particularly with respect to transmission routes and effective long-term management strategies.
